# Supplementary material for: Rapid turnover of life-cycle-related genes in the brown algae
Source: Genome Biol. 2019 Feb 14;20:35. doi: 10.1186/s13059-019-1630-6 (PMC6374913; doi:10.1186/s13059-019-1630-6)
Supplement: Supplementary file 3 — Protein alignments, corresponding Gblocks html file and CDS sequences for the lineage conserved gametophyte-biased genes. (ZIP 2109 kb) [file 13059_2019_1630_MOESM3_ESM.zip › Con_GA_alignments/Ec-26_002230.aa_ali.fasta-gb.htm]

Ec-26\_002230.aa.fasta.trans.aa\_ali.fasta


## Gblocks 0.91b Results

Processed file: **Ec-26\_002230.aa.fasta.trans.aa\_ali.fasta**  
Number of sequences: **2**  
Alignment assumed to be: **Protein**  
New number of positions: **207** (selected positions are underlined in blue)

```
                         10        20        30        40        50        60
                 =========+=========+=========+=========+=========+=========+
Ec-26_002230     -----------------------------MAASIDSPAWTFGLNPTVPGPRVPGPGTYNV
MSTRG.33277.1    PPHISTRLYTPLPLDAATASDAIRFNAVRMAIGVDSPAWTFGLNPKIPGPRVPGPGTYDV
                                              ###############################


                         70        80        90       100       110       120
                 =========+=========+=========+=========+=========+=========+
Ec-26_002230     AGSLDGGSAGTFFSPIGAKPTCPRTDVKRLVFGIEREGRDKPGPGAYDVTKNRP-LRWNV
MSTRG.33277.1    AGRLGVGSVGTRFTPVGAKPASPRTDGTRLIFNTEEENRQKPGPGAYDLIEQRPRRRCNV
                 ######################################################      


                        130       140       150       160       170       180
                 =========+=========+=========+=========+=========+=========+
Ec-26_002230     SP-RDRKHQTPRFPVSSPATDAGPGAYISSEFAGKGPAYSMPSRSPTTLDTPESPGPGQY
MSTRG.33277.1    RPPGGTNTETPRFPESSHATDAGPGAYIPSEFASGGPAYSLPAREPVALDAPASPGPGQY
                          ###################################################


                        190       200       210       220       230       240
                 =========+=========+=========+=========+=========+=========+
Ec-26_002230     VHVQLSVGRGENGAGEGFGKRDSPRFATEATPTPGPGEYQSPAFNPQLGKNITFGRNLRP
MSTRG.33277.1    IEVHLSATPDVTDSSEGFGRRDSPRFDDEPAQSPGPGAYQSARFCPQLGKNMTFGGPFRP
                 ######         #############################################


                        250       260       270       280       290       300
                 =========+=========+=========+=========+=========+=========+
Ec-26_002230     AGWSARIKEITRFACELDGDNDDDDDCDGDQQWSGGWRRVGRFRAPLHHHRRESAERETI
MSTRG.33277.1    PGWSAKIKEITRFACELDDD----------------------------------------
                 ####################                                        


                        310       320       330       340       350       360
                 =========+=========+=========+=========+=========+=========+
Ec-26_002230     IARLRRRPSSAPPSSRFRRACARGRRLDRTADGGGGGGDRTEKKQQRGGTANGSSSSSST
MSTRG.33277.1    -----------------------------------------------GSNGNGS------
                                                                             


                        370       380       390       400       410       420
                 =========+=========+=========+=========+=========+=========+
Ec-26_002230     CRRPRFIADVVKEAGRRKRAKSAAAMSAKITGVGASPSKRRSRETATGRGSSMGTAPQRP
MSTRG.33277.1    ------------------------------------------------------------
                                                                             


                        430       440       450       460       470       480
                 =========+=========+=========+=========+=========+=========+
Ec-26_002230     PDAAPSPGPSSYLPATLDETARDGKVVGIGGRSKDSAPRPGVGDYDLRAAEAAAAGSSTA
MSTRG.33277.1    ------------------------------------------------------------
                                                                             


                        490       500       510       520       530       540
                 =========+=========+=========+=========+=========+=========+
Ec-26_002230     VPPTLGRKPTGAPYDPALSRYLFRDDGRVTPSAHAYRPESATGGGGDGGLAGQAAAPPAW
MSTRG.33277.1    ------------------------------------------------------------
                                                                             


                        550       560       570       580       590       600
                 =========+=========+=========+=========+=========+=========+
Ec-26_002230     SFGGKRPTPRPPDGPGPGKFRPPYNNETDPCCSSSPAGVRFGAGREDMSGSVSGVPWVEA
MSTRG.33277.1    ------------------------------------------------------------
                                                                             


                        610       620       630       640
                 =========+=========+=========+=========+====
Ec-26_002230     EERAARGEAGGRSARLYLPERDRMGSRAFMRSRQLPRPGTRGPR
MSTRG.33277.1    -------------------------------------------X
```

```
Parameters used
Minimum Number Of Sequences For A Conserved Position: 2
Minimum Number Of Sequences For A Flanking Position: 2
Maximum Number Of Contiguous Nonconserved Positions: 8
Minimum Length Of A Block: 10
Allowed Gap Positions: None
Use Similarity Matrices: Yes
```

```
Flank positions of the 3 selected block(s)
Flanks: [30  114]  [130  186]  [196  260]  

New number of positions in Ec-26_002230.aa.fasta.trans.aa_ali.fasta-gb:  207  (32% of the original 644 positions)
```
